# Comparing Iliac Artery Sizes to Explain Post-EVAR Non-Obstructive Thrombosis. Comment on Lee et al. Influence of Aortoiliac Geometry on Non-Occlusive Thrombotic Risk Following Endovascular Repair of Abdominal Aortic Aneurysms. *Diagnostics* 2025, *15*, 2134

**DOI:** 10.3390/diagnostics15202551

**Published:** 2025-10-10

**Authors:** Wouter Kok

**Affiliations:** Department of Clinical and Experimental Cardiology, Amsterdam Cardiovascular Sciences, Meibergdreef 9, 1105 AZ Amsterdam, The Netherlands; w.e.kok@amsterdamumc.nl

In the paper by Lee et al., ‘Influence of Aortoiliac Geometry on Non-Occlusive Thrombotic Risk Following Endovascular Repair of Abdominal Aortic Aneurysms’, the conclusion was drawn that ‘anatomical characteristics such as larger iliac diameters, decreased vessel angulation, and increased tortuosity were identified as key risk factors for post-procedural thrombosis’ [1]. The conclusion resulted from a comparison between thrombotic iliac arteries (*n* = 18) and non-thrombotic iliac arteries (*n* = 90), with maximal iliac artery diameters of 17.48 ± 0.95 mm versus 14.14 ± 0.62 mm, respectively. What the authors did not publish was asked in the review process, a comparison between the absolute diameters of the left and right iliac arteries after EndoVascular Aneurysm Repair (EVAR) in 36 patients without thrombotic iliac arteries. This revealed that the maximal diameters of the left and right iliac arteries (18.5 ± 1.3 versus 17.1 ± 0.7 mm, respectively) were as large as those of the thrombosed iliac arteries. This result would indicate that it was not the absolute maximal iliac diameter after EVAR that may have predisposed to thrombosis, but that it was more the relative size of the maximal iliac diameter compared to that patient’s iliac diameter size on the other side that makes the difference. When interpreting iliac artery size, the comparison with a ‘normal’ size that is appropriate for the patient is not easy, there seems to be no standardized size. However, for this reason a paired comparison within the patient’s iliac arteries is more appropriate than an unpaired comparison with varying sizes of iliac arteries. Even then, the abnormality in diameter or other factors associated with iliac artery thrombosis after EVAR should not be present as often in those without the thrombosis. The implication of this comment is that it is more likely that in the study by Lee et al. it were patients with smaller iliac artery diameters who would be predisposed to iliac artery thrombosis after EVAR, for example by oversizing the iliac artery. Or it may be explained by a pre-intervention aneurysm of the predisposed iliac artery. The finding that patients with smaller iliac diameters before intervention are predisposed to EVAR complications has been demonstrated before in a study by Draper et al., which the authors also have referenced [2]. For the author’s sake and also for future studies on this problem, a comparison between preintervention and postintervention dimensions would be required to better understand the process of iliac artery thrombosis after EVAR.
